# Factors associated with behavioral euthanasia in pet dogs

**DOI:** 10.3389/fvets.2024.1387076

**Published:** 2024-04-17

**Authors:** Miranda Hitchcock, Miranda K. Workman, Adeline P. Guthrie, Audrey Ruple, Erica N. Feuerbacher

**Affiliations:** ^1^School of Animal Sciences, Virginia Polytechnic Institute and State University (Virginia Tech), Blacksburg, VA, United States; ^2^Department of Animal Behavior, Ecology, & Conservation, Canisius University, Buffalo, NY, United States; ^3^Department of Statistics, Virginia Polytechnic Institute and State University (Virginia Tech), Blacksburg, VA, United States; ^4^Virginia-Maryland College of Veterinary Medicine, Department of Population Health Sciences, Virginia Polytechnic Institute and State University (Virginia Tech), Blacksburg, VA, United States

**Keywords:** euthanasia, behavior problems, behavioral euthanasia, canine behavior, aggression, dogs, behavior, behavioral problems

## Abstract

When pet dogs demonstrate certain serious problem behaviors, this may lead owners to choose behavioral euthanasia. However, research on behavioral euthanasia of pet dogs is sparse and previously published papers have not specifically sampled owners who made the decision to euthanize for behavior. The Behavioral Euthanasia in Pet Dogs Questionnaire was created to collect a wide range of information from dog owners who made behavioral euthanasia decisions. Using this survey, we explored the types of behaviors associated with behavioral euthanasia. Human-directed aggression, especially toward adults living in the household, was the most frequently reported reason for choosing behavioral euthanasia; followed by aggression toward other animals, especially other dogs living in the same household. The majority of dogs displaying human-directed or other animal-directed aggression were reported to have bitten and broken skin, and many of these had bitten in multiple or severe incidents. Most dogs had lived in their homes and displayed problem behaviors for over a year prior to behavioral euthanasia, and the euthanasia occurred at a variety of ages, from less than 1 year old to 18 years old. Additional research is required to understand environmental or nonbehavioral factors contributing to the behavioral euthanasia of companion animals, as well as the psychosocial and emotional impact of behavioral euthanasia on the human experience. Understanding the behavioral factors associated with behavioral euthanasia can direct resources toward problem behavior interventions, improve public education about animal behavior, and strengthen the human-animal bond.

## 1 Introduction

According to the American Pet Products Association, 66% of U.S. households include companion animals (1). Owners are estimated to spend over $143 billion dollars caring for their companion animals in 2023 (1). A majority of these households (75%) have dogs, and those dogs are considered to be “family” by 86% percent of owners (2–5).

There are a number of potential challenges of living with and caring for dogs (6, 7). Dealing with problem behaviors can be one of those challenges, as demonstrated by studies reporting that 40–92% of dogs present with behavior issues (8–16). Understanding the occurrence and prevalence of problem behaviors in pet dogs can be challenging due to the diverse range of descriptions, terminology, and categorization used in research (17–20). For example, the broad single category of “aggression” has included barking, non-injurious bites during play, and even injurious or fatal bites across studies (16, 21, 22). Within the category of aggression, researchers have classified aggressive behaviors by victim, circumstance or trigger, and motivation (17, 23–26).

When pet dogs display problem behaviors, this can result in negative consequences for the community, the human-animal bond, the owner, and the dogs themselves (16, 21, 27–35). Poor human-animal relationships and euthanasia decisions can also negatively impact professionals such as dog behavior consultants and veterinarians (36).

If behavior problems are seen as severe by the dog’s owners, such as aggression, owners might seek assistance from veterinary and animal behavior professionals or choose to remove the dog from their home by rehoming them to another household or relinquishing them to an animal shelter or rescue (17, 19, 26, 37–39). When considering these negative consequences (such as having to seek professional help or rehoming the dog), aggressive behavior was identified as the most commonly reported problem behavior by multiple studies (17, 19, 23, 26, 37, 40).

Problem behaviors that increase the risk of harm or decrease welfare for the dog or others around them may result in euthanasia of the dog, despite the dog being physically healthy (41, 42). This is commonly known as behavioral euthanasia (BE). Few studies have attempted to identify the prevalence or risk factors for behavioral euthanasia in owned dogs (28, 43–55). Several of these studies have focused on prevalence at a population level and have not delved into the specifics of the behaviors exhibited by those dogs, while others looked at risk factors within a smaller subset of dogs.

Despite the limited research on this topic, dog behavior practitioners are drawing attention to this area through conferences, online courses, articles, and blogs (56–65).

Previous research suggests that the primary behavioral cause for euthanasia is aggression. In one study that investigated why dogs were surrendered for behavioral reasons to an animal shelter, (47) found that the most common reasons were human-directed aggression, bites to people or animals, or animal-directed aggression. This is in agreement with other studies that indicate aggression, particularly aggression toward people, is a major factor in behavioral euthanasia decisions (28, 43–46, 48–55).

However, the existing studies do not investigate these behaviors in-depth to evaluate commonalities in severity levels or details of aggression. Currently, practitioners must rely largely on anecdotal evidence when it comes to the behaviors most commonly associated with euthanasia in pet dogs. Therefore, the purpose of this study was to identify which specific behavioral factors in dogs were associated with behavioral euthanasia decisions by owners using the Behavioral Euthanasia in Pet Dogs Survey.

## 2 Materials and methods

### 2.1 Subjects

Subjects were primarily recruited online from social media groups focusing on behavioral euthanasia, dog behavior, or dog ownership. Additional recruitment strategies included email list-servs and emails with veterinary behaviorists and trainers. The resulting sample is one of convenience rather than a statistically derived sampling method. Participation in the study was limited to adults in the United States who had euthanized a pet dog due to behavior problems any time on or after January 1st, 2017. The dog must have been owned by the respondent, not a dog in a shelter or foster environment.

In order to minimize potential recall bias, the sample was limited to those who had euthanized a dog for behavior concerns within approximately the 5 years immediately prior to survey completion (66).

### 2.2 Questionnaire

We designed a questionnaire to collect a broad range of information about dogs that were euthanized for behavior-related issues. Due to the exploratory nature of this research, only a portion of the questions used in this questionnaire could be obtained from validated, previously published questionnaires. For example, several questions about the euthanized dog and its living situation were adapted from questionnaires used in the Dog Aging Project (67). Human demographic information questions were retrieved from the 2020 U.S. Census (68). The remaining questions were written based on the consensus opinion of more than 20 experts in dog behavior. These experts included board certified veterinary behaviorists, animal behavior researchers, certified dog behavior consultants, and dog trainers who specialize in complex cases.

The resulting Behavioral Euthanasia in Pet Dogs questionnaire was designed to focus on simple descriptions and lay terminology wherever possible. This limits potential differences in responses based on level of behavioral education. Questions used situational descriptions such as whether the dog showed aggression when “approached while eating,” vs. potential labels or causes for that behavior (e.g., dominance or resource guarding).

The questionnaire consisted of 118 questions designed to cover a wide range of information about dogs that were euthanized for behavioral reasons. Respondents were asked for basic signalment and information about the dog, their living situation, and their acquisition. They then ranked up to three categories of behavior as being the primary reasons for the euthanasia (aggression toward people; aggression toward dogs or other animals; fear, anxiety, or stress; compulsive behaviors; separation anxiety; and other behaviors). These ranked categories determined which survey sections respondents would see later in the survey, with branching logic showing the respondent detailed behavior questions related to the behaviors they had listed. The questionnaire also included questions about treatments or interventions respondents had attempted prior to euthanasia as well as non-behavioral factors that contributed to the euthanasia decision.

Behavior questions were written based on results and methods from previous research as well as anecdotal experience from behavior experts (28, 43–55). These questions include information about types of behaviors, specific targets or circumstances for those behaviors, the severity involved, and any precursors to those behaviors.

In order to understand comorbid behaviors and patterns, most questions allowed respondents to select as many responses as applied. Thus response count (total number of responses submitted by all respondents) and respondent count (total number of respondents who answered that question) are listed throughout.

This research project was approved by the Virginia Tech Institutional Review Board, with exemption under 45 CFR 46.104(d) category (ies) 2(i) with IRB number 22–311. The first page of the survey included a consent form.

Summary descriptive statistics were calculated using JMP Pro Version 16.1.0 (69) (RRID: SCR_022199). All figures were created using R (70) [RRID: SCR_001905; (71), RRID: SCR_014601; (72), RRID: SCR_024824; (73), RRID: SCR_024825; (74), RRID: SCR_024826].

## 3 Results

We recruited a total of 800 respondents who met the selection criteria. Of these, 729 completed the questions about dog signalment and sourcing, and 690 completed the ranking of primary behaviors that led to the decision to euthanize. Finally, 575 respondents completed the entire survey through the demographic questions at the end. Data analysis of each question included all responses to that question; therefore, the response count varies by question (see Supplementary Figure for details).

Of the 575 respondents who provided demographic information, 94.5% identified as women and 91.3% identified as White, Non-Hispanic. Respondents ranged in age from 18 to 65+ years old, but over one third of all respondents reported being ages 25–34 at the time of the dog’s euthanasia. Almost two thirds of respondents reported having a bachelor’s degree or higher level of education or training. At the time of euthanasia, 34.1% of respondents had a household annual income of $50,000–$99,000. The next most common income range was $100,000–$199,000 (29.1%), followed by under $50,000 per year (21.2%).

The respondents reported on 729 dogs, which included 69.4% males and 30.6% females, with 89.2% of all dogs being spayed or neutered. These dogs ranged from three kilograms to 102 kilograms in weight, with a median of 27 kilograms and a mean of 28 kilograms. Over half of the dogs were acquired prior to 1 year of age.

### 3.1 Primary behavior problems that led to euthanasia

The “Primary Behaviors” question in the Behavioral Euthanasia in Pet Dogs questionnaire asked respondents to rank up to three behavior categories that contributed to their behavioral euthanasia decision. In this study, 690 respondents completed the survey section that provided information on the primary, secondary, and tertiary behavioral reason for euthanasia.

**Figure 1:**
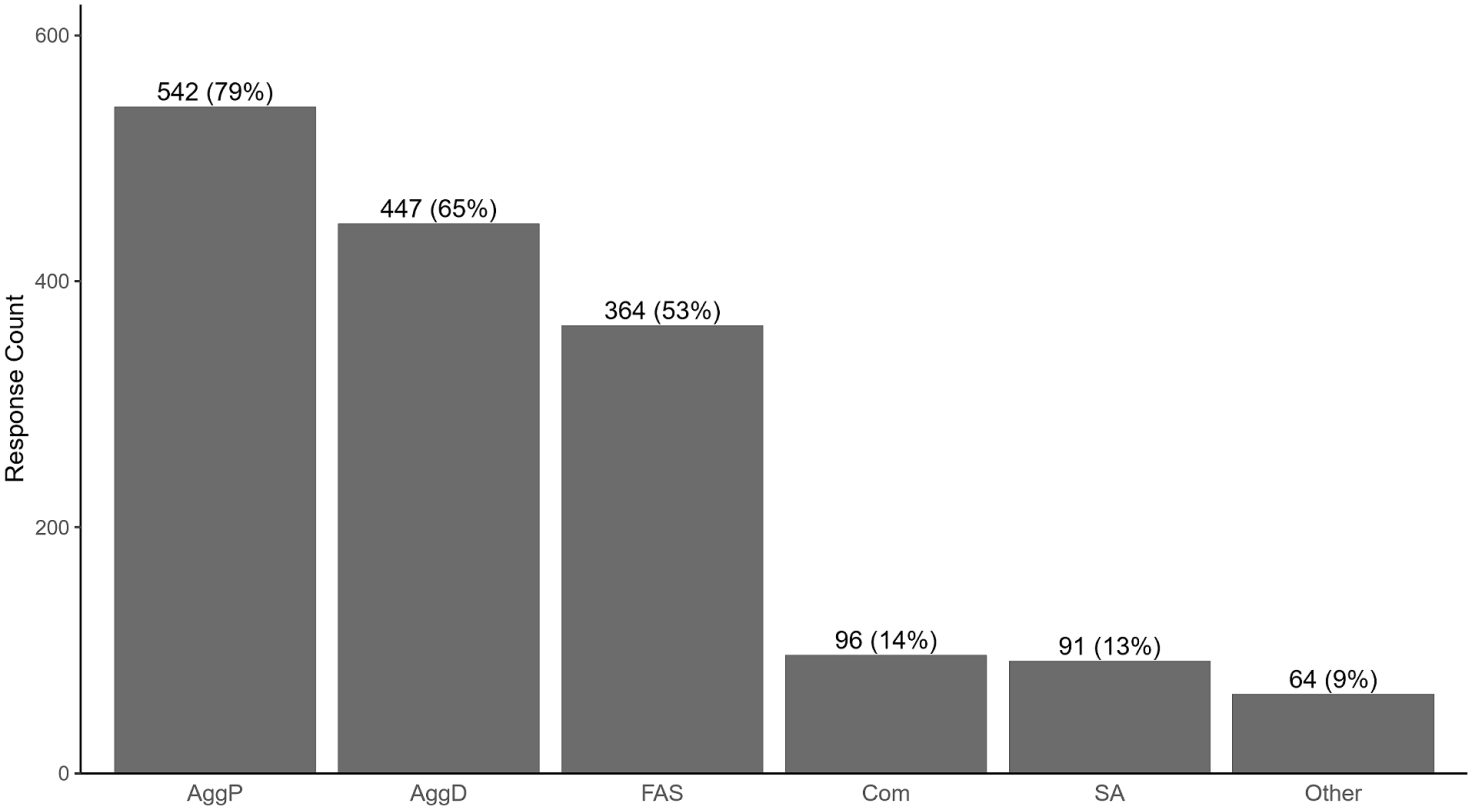
Primary behavior categories that led to owners’ behavioral euthanasia decision. Legend: average rank: aggression toward people 1.33, aggression toward animals 1.73, fear, anxiety, or stress 2.17, separation anxiety 2.35, compulsive behavior 2.31, other 2.53. Total number of responses (regardless of rank) by behavior type. Total response count = 1,604, total number of respondents *n* = 690. Respondents could rank up to three behaviors. Percentage represents portion of respondents (690), not portion of responses.

Aggression toward people was the most commonly reported behavior category at 33.8% of all responses (78.6% of respondents), followed by aggression toward dogs or other animals with 27.9% of responses (64.7% of respondents; Figure 1). Fear, anxiety, and stress accounted for 22.6% of responses (52.8% of respondents), with separation anxiety and compulsive behaviors represented in 6.0 and 5.7% of responses, respectively, (13.9 and 13.2% of respondents). Only 4.0% of responses were for the “Other” category of behavior (9.0% of respondents).

Of the 19.7% who only chose a single behavior, 63.2% of responses were aggression toward people and an additional 30.2% were aggression toward dogs or other animals. There were only six respondents who selected fear, anxiety, or stress as the only problem behavior leading to euthanasia. Two respondents chose “Other,” and one chose compulsive behavior.

**Figure 2:**
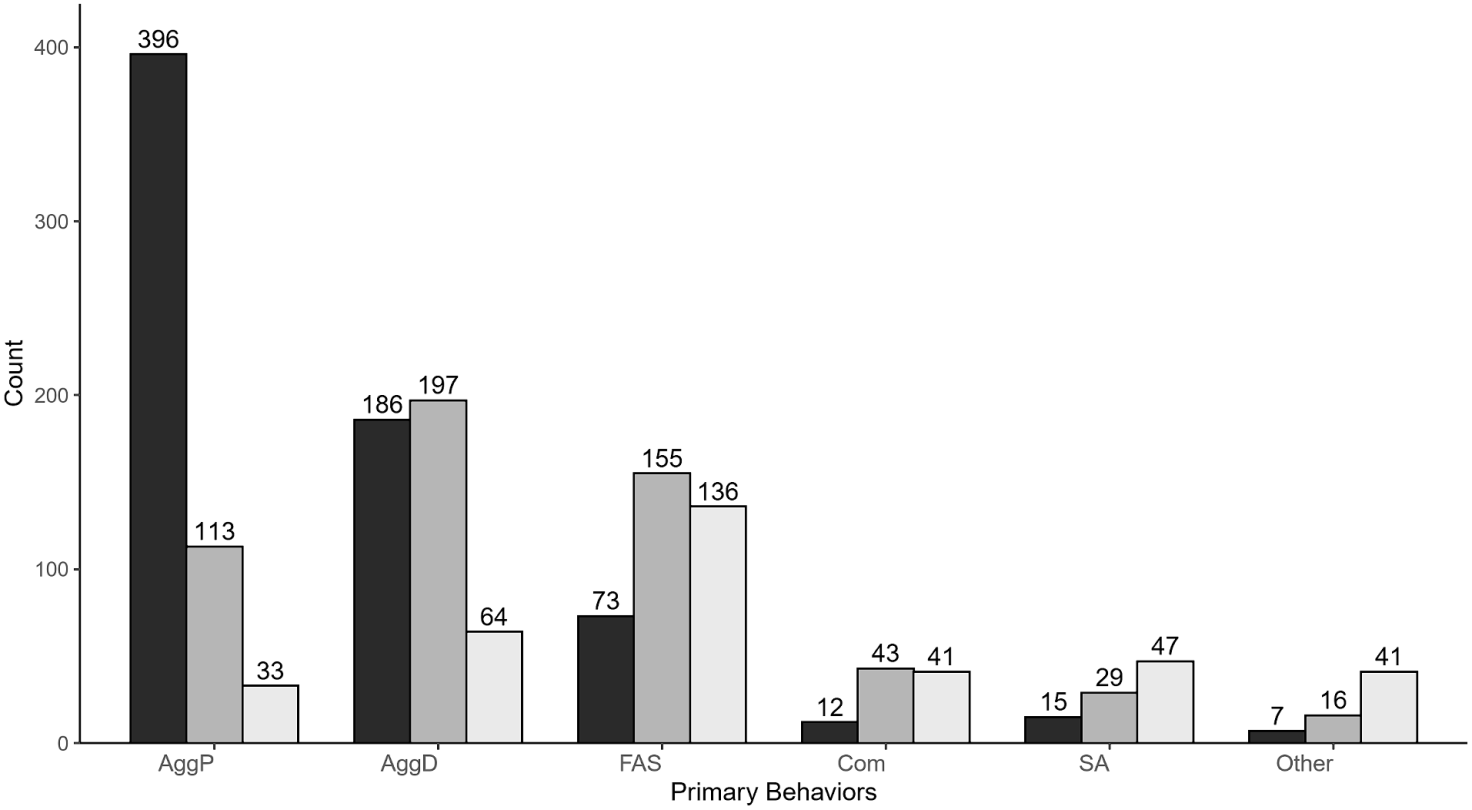
Problem behaviors leading to behavioral euthanasia, ranked as primary, secondary, and tertiary reasons. AggP, aggression toward people. AggD, aggression toward dogs or other animals. FAS, fear, anxiety, or stress. SA, separation anxiety. Com, compulsive behavior. Legend: Rank: 1st (black), 2nd (medium gray), 3rd (lightest gray). Total response count = 1,609, total number of respondents *n* = 690.

In addition to calculating total number of responses per category, we also separated them by participants’ ranking (first, second, or third most problematic; Figure 2). Aggression toward people was the most highly ranked as a problematic behavior, with an overall average ranking of 1.33/3 ignoring non-rankings. It was listed as the primary behavior that led to a euthanasia decision by 57.4% of all respondents and was ranked in the top three factors by 78.6% of all respondents. Of the 542 respondents who included aggression toward people as one of their top three reasons for euthanasia, 73.1% marked it as the number one cause, with 20.8% marking it second and 6.1% marking it as the third most important factor.

While aggression toward people was commonly ranked as the most important reason for euthanasia, categories like fear, anxiety, and stress or compulsive behaviors were more likely to be ranked second or third. Of those who marked fear, anxiety, or stress as one of their ranked behaviors, only 20.0% ranked it first vs. 42.6% who ranked it second and 37.4% who ranked it third.

Comorbidities were common among the various categories of behavior, with 80.1% of respondents selecting more than one primary behavior. The most common combinations of categories were aggression toward people followed by aggression toward animals, with 26.8% of respondents choosing this option. The next most common combination was aggression toward people followed by fear, anxiety, or stress (12.9% of respondents).

### 3.2 Time in the home, age, and onset of behavior problems prior to euthanasia

**Figure 3:**
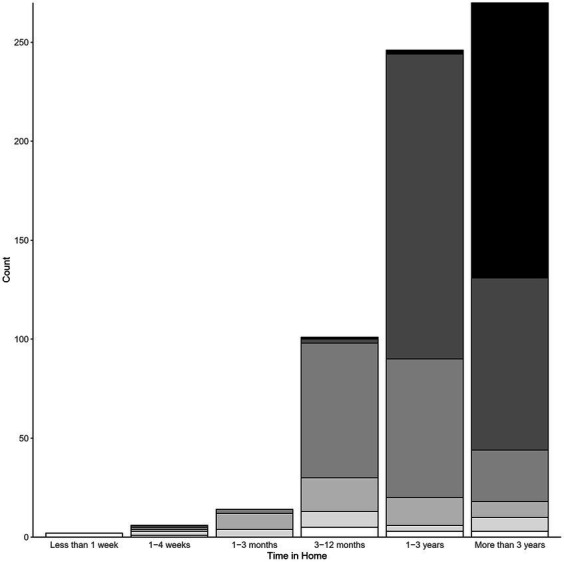
Time since onset of problem behavior (s) by length of time in home at time of behavioral euthanasia. Legend: time since onset (from lightest color to darkest): less than 1 week, 1–4 weeks, 1–3 months, 3–12 months, 1–3 years, More than 3 years. *n* = 639.

The majority of dogs had been in the home for at least a year prior to euthanasia, and 42.3% had been in the home more than 3 years. Additionally, most dogs began displaying the problem behavior at least 1 year prior (60.4%, Figure 3). Twenty six percent of dogs had only shown the behavior for 3–12 months prior to euthanasia, and only 5.9% of dogs began the behavior less than 1 month before.

**Figure 4:**
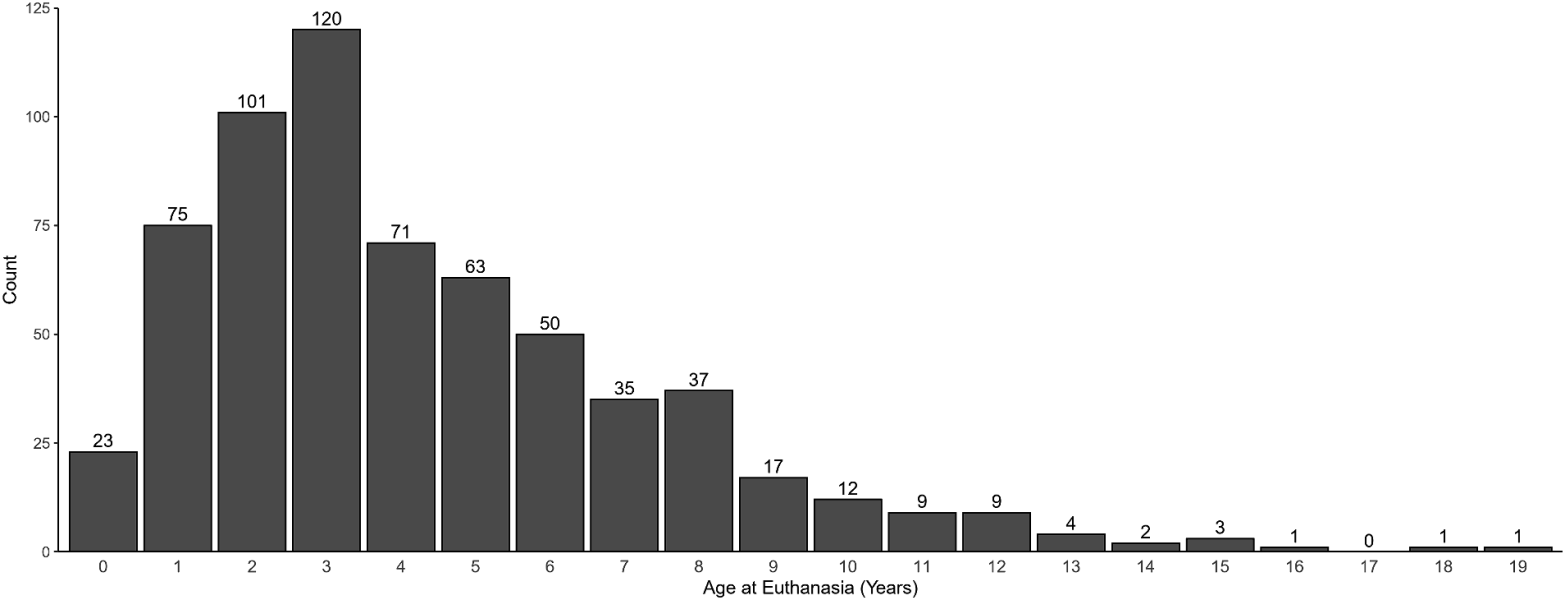
Age of the dog at time of behavioral euthanasia. *n* = 634, mean age = 4.31 years, SD = 308.

The dogs represented in this study were euthanized at ages ranging from under 1 year old to 18 years old, with a median of 3 years old and a mean of 4.31 years old (Figure 4). Only 3.6% of dogs were less than 1 year old at euthanasia, and 6.6% were 10 years old or older. Of those older dogs, 81.0% had been in the home for at least 3 years. However, the onset of problem behaviors within all of the older dogs was varied. Of these dogs that were euthanized at age 10 or older, 42.9% had demonstrated the problem behavior (s) for at least 3 years. However, only 21.4% had shown it for one to 3 years and another 23.8% demonstrated the problem behavior from three to 12 months. The remaining 11.9% of dogs 10 or older only began exhibiting the behavior issue less than 3 months prior to euthanasia.

In addition to questions about age and onset of behavior problems, respondents were asked to identify whether there was a single behavioral incident or final incident (compared to no specific incident) that contributed to the behavioral euthanasia decision. The majority of respondents indicated that behavioral euthanasia was based on a final incident (77.9%), vs. 5.8% indicating a single incident and 16.3% reporting that no specific incident led to the decision.

### 3.3 Aggression toward people

#### 3.3.1 Targets

**Figure 5:**
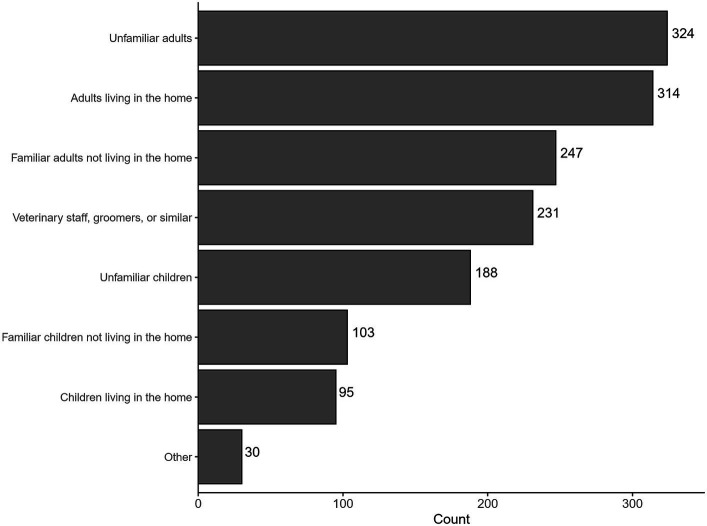
Total responses for potential targets of aggression toward people. Total response count 1,532. The 476 respondents who said that their dog’s aggression toward people was one of the primary reasons for euthanasia were able to choose as many categories as applied.

Of the 542 respondents who selected aggression toward people as a primary behavior leading to the decision to euthanize, 476 responded to questions about the types of targets of the dog’s aggression. These types included familiar adults or unfamiliar children, for example, and respondents were able to choose multiple targets of aggression (Figure 5). Most respondents (83.8%) selected multiple targets of aggression, with a mean of 3.2 categories of people the dog aggressed toward. The remaining 16.2% selected a sole target for the dog’s aggression toward people. Of these 16.2%, over half reported aggression solely toward adults in the home.

Aggression solely toward adults in the home was more prevalent than expected: 10.3% of all respondents who answered the section about aggression toward people (n = 476) indicated that the dog was only aggressive toward adults in the home. The next most common response pattern (7.4%) was aggression toward all adults, followed by 4.6% reporting all options (children and adults).

Aggression solely toward children was rare, reported by only 2.7% of respondents. Even more uncommon was aggression specifically to a single category of child, with 1.9% of respondents marking children in the home as the target of aggression and 0.4% noting familiar children not living in the home as the sole target.

#### 3.3.2 Predictability and triggers

Respondents were asked about their ability to predict their dog’s aggression toward people as well as their ability to notice warning signals prior to aggression. Forty two percent of respondents indicated that they could predict aggressive reactions “most of the time” or “always,” while only 30.9% of respondents in this section said the dog showed clear warning signals “most of the time” or “always.” However, both categories demonstrated wide variation ranging from “always” to “never.”

Respondents also reported the scenarios or triggers that led to aggression toward people. On average, respondents chose 5.12 of the 15 listed circumstances, with only 12.6% of the 476 respondents selecting a single trigger. The most common circumstances included being approached by an unfamiliar person while on leash (9.3% of respondents) or when a person moved quickly or erratically (8.8% of respondents). Other common responses were when delivery workers approached the house or when being touched or handled (7.7% each).

#### 3.3.3 Bites and severity

**Figure 6:**
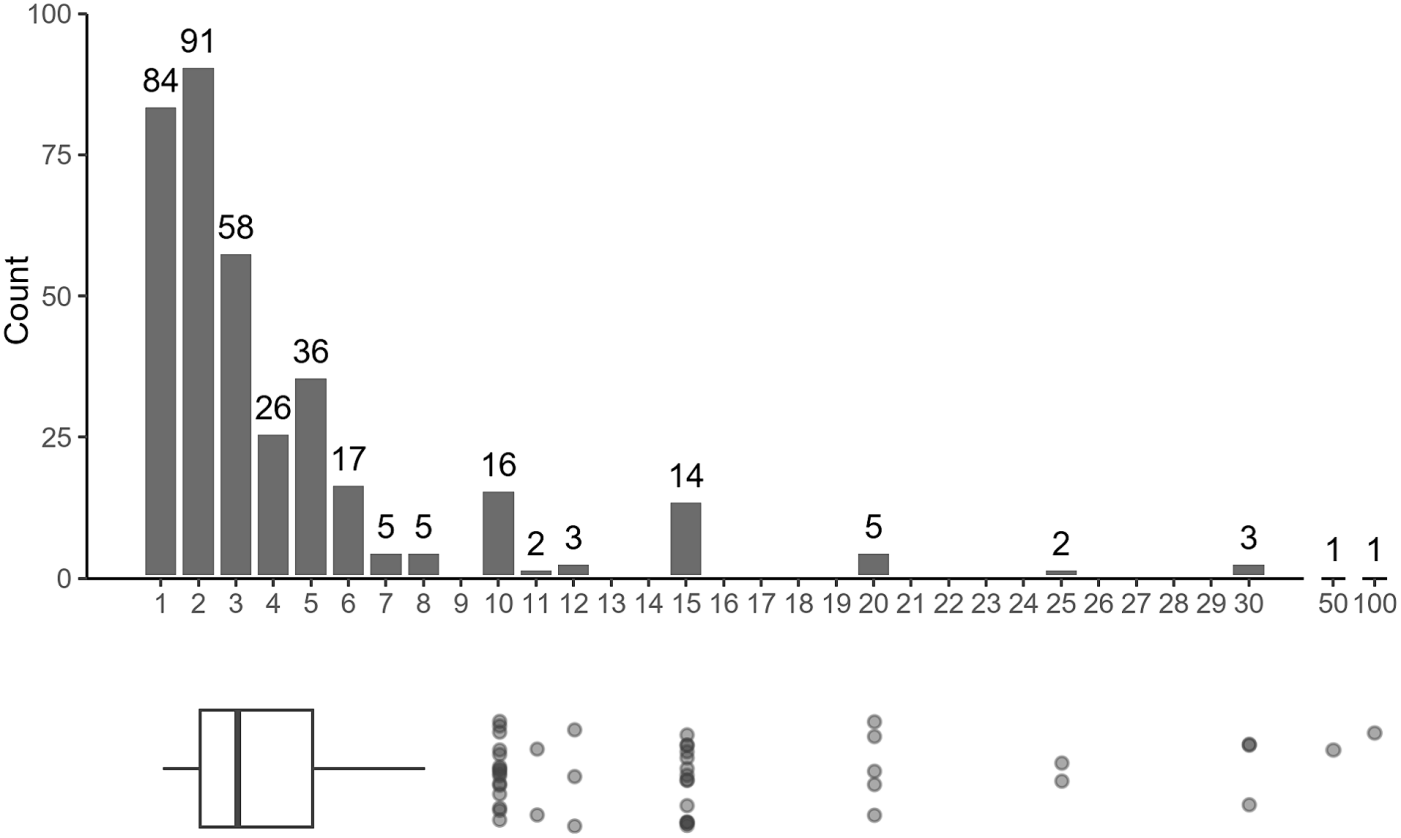
Number of skin-breaking bites reported per dog with aggression toward people. *n* = 369 and the two highest outliers (50 bite and 100 bites) were broken from the main axis for better visual representation of the main data. The upper and lower limits of the box denote the 25 and 75th percentiles at two bites and five bites, respectively. The median was three bites. The whiskers show the lower and upper limits of the observed values (excluding outliers) at one bite and eight bites, respectively.

Of the respondents that indicated their dog showing aggression toward people, 77.5% reported that the dog had bitten and broken skin on a person at least once. The median number of bites was three, and outliers reported as many as 50 to 100 skin breaking bites (Figure 6). Of the 369 dogs who had broken skin, 22.8% had bitten only once. However, almost 36.9% of biting dogs had bitten people at least four times, and 12.7% were reported to have bitten 10 or more times.

If respondents indicated their dog had bitten a person and broken skin, they were asked to describe the severity of the inflicted bite wounds (Table 1). Respondents were able to select multiple wound types based on the bites their dog had inflicted. The most common wound type, accounting for 70.5% of total responses, was “1–4 small punctures from a single bite.” While 37.4% of bites were the least severe type noted above, an additional 18.4% of responses were small tears or lacerations. However, 41.9% of reported bites were more severe and would likely involve multiple bites within the same incident and/or require stitches. Of the reported bites, 29.1% would be considered most severe (including nine or more punctures, multiple or large tears or lacerations, or crushing injuries and broken bones).

**Table 1:** Types of wounds inflicted in skin-breaking bites to people by dogs with aggression toward people.

Injury Type	Severity	Responses (count)	Responses (percent)	Worst bite per dog (count)	Worst bite per dog (percent)
1–4 small punctures from a single bite.	Least Severe	260	37.4	122	33.1
1–3 small tears or lacerations (up to ½ inch).	Moderately Severe	128	18.4	70	19.0
5–8 punctures from the same incident	Moderately Severe	89	12.8	31	12.8
Tears or lacerations greater than ½ inch, or fatty tissue, muscle, etc. exposed.	Most Severe	94	13.5	79	21.4
More than 3 tears or lacerations from multiple bites in the same incident	Most Severe	59	8.5	26	7.0
Crushing injuries or broken bones	Most Severe	31	4.5	31	8.4
9–15 punctures from the same incident	Most Severe	13	1.9	3	0.8
16+ punctures from the same incident	Most Severe	5	0.7	0	0.0
Other	Unknown	9	1.3	2	0.5
I do not know	Unknown	8	1.1	5	1.4

Total response count = 696. Number of respondents reporting that their dog had bitten a person and broken skin = 369. Response Count column represents all responses, and the following column shows the percentage of the 696 overall responses.

In addition to assessing the overall prevalence of different types of bites, we analyzed the data to look at the most severe bite per dog. If a respondent indicated several types of bites, the category of their worst bite was recorded. When only looking at the highest severity level per dog, 37.6% of dogs had a bite in the “most severe” category.

While wounds were reportedly treated at home without professional medical attention 35.9% of the time, 10.8% of all reported bites required more than 10 stitches, other surgical intervention, or hospital admission. When considering just the worst reported bite per dog, the percent of bites requiring the aforementioned types of extensive medical treatment rises to 13.8%.

### 3.4 Aggression toward dogs or other animals

#### 3.4.1 Targets

Aggression toward dogs or other animals was the second most commonly reported problem behavior that led to behavioral euthanasia, selected by 447 participants; 394 of these respondents answered the detailed questions pertaining to that aggression. Aggression toward dogs made up the majority of animal-directed aggression, with 62.7% of those 394 respondents reporting that their dog was only aggressive to other dogs, not to other animals. Overall, aggression toward dogs was reported by 76.7% of respondents who said their dog showed aggression toward animals. Among dogs that showed aggression toward other animals, the most common target of that aggression was another dog (or dogs) in the home (31.2% of 394; Table 2). The next most common targets were unfamiliar dogs (27.9%) and familiar dogs not living in the home (17.6%). The most common responses/combinations for this section were either dogs in the home (18.1% of respondents), unfamiliar dogs (10.7% of respondents), or a combination of the two (11.0%).

**Table 2:** Total responses of types of targets for dogs’ aggression toward other dogs or other animals.

Target/Victim	Response count	Percent of total responses (1486)	Percent of respondents (394)
Other dogs in the home	283	31.2	71.7
Unfamiliar dogs	253	27.9	64.1
Familiar/known dogs not living in the home	159	17.6	40.3
Unfamiliar cats	101	11.2	25.6
Cats in the home	81	8.9	20.5
Livestock	9	1.0	2.3
Small wildlife	7	0.8	1.8
Unspecified/ “all animals”	5	0.6	1.3
Large wildlife	4	0.4	1.0
Chickens	4	0.4	1.0

Response count = 906, with 394 respondents. Livestock, all wildlife, and chickens were aggregated within the “Other” category.

Of the respondents who reported their dog showing aggression toward other animals (n = 394), 133 indicated a single type of target of aggression. Of those 133, 66.2% aggressed toward other dogs in the home and 27.1% aggressed toward unfamiliar dogs. Only five respondents listed aggression toward cats in the home as the sole aggression toward other animals. No other targets (such as wildlife, unfamiliar cats, or livestock) were listed as sole choices, instead only being reported in combination with aggression toward dogs.

#### 3.4.2 Triggers

Respondents were asked to indicate what circumstances or triggers would cause aggression toward other animals (Table 3). While the responses varied widely, the most commonly reported triggers were: being approached while on leash (10.4%), an escalation in play/excitement (9.5%), being approached while engaging with a preferred object like a toy or a bone (9.4%), and predatory behavior (9.4%).

**Table 3:** Environmental stimuli/circumstances for aggression toward dogs or other animals.

Circumstances	Count	Percent
Being approached directly by the other animal while the dog was on leash	154	10.3
Escalation in play/excitement	141	9.5
Being approached while playing with/chewing on a favorite toy, bone, or other object	140	9.4
Predatory behavior (attempting to hunt or prey on the other animal)	140	9.4
When an animal entered the dog’s yard or home	137	9.2
When a preferred person was approached by the other animal	126	8.5
Being approached in/on a preferred area, such as a couch or bed	119	8.0
Being approached while eating	111	7.5
A “redirection” where the dog was reacting to something else but bit the other animal instead, for example when the dog was reacting on leash or running along the fence	88	5.9
Other	58	3.9
When woken up from sleeping or startled	57	3.8
I do not know	53	3.6
Joined in when other dog(s) were already fighting	49	3.3
The other animal attacked, cornered, or otherwise threatened the dog	39	2.6
None of the above	27	1.8
The dog itself was injured, sick, etc.	26	1.8
Changes in health or appearance of the other animal	22	1.5

Response count = 1,486, with 394 respondents marking as many responses as applied. Percent of responses looks at the instances among all 1,486 responses, while percent of respondents is out of the 394 respondents.

#### 3.4.3 Bites and severity

Of the 394 dogs that were reported to aggress toward other animals, 69.5% had previously bitten an animal and broken skin. Of the dogs that had bitten, 16.4% had done so only once. Another 21.2% had broken skin twice, with 19.6% biting three times. Almost 44% of dogs that had previously bitten had broken skin four times or more, while 13.9% had done it at least 10 times.

Of the skin-breaking bites to other animals, 29.4% were a single bite with 1–4 small punctures (Table 4). When looking at the dogs that had bitten (*n* = 274), 10.9% of respondents in this category reported that the other animal had been killed from the bite incident, and 6.6% said the other animal was wounded badly enough to be euthanized. Upon closer examination of the instances where the dog either fatally attacked another animal or caused injuries severe enough to necessitate euthanasia, the predominant factor was again aggression toward dogs. The other animals in these cases included dogs (18), cats (4), and wildlife (3) based on the free text responses, while other participants did not note the type of animal. According to notes provided by respondents, one dog was responsible for the deaths of two cats and another dog in the household. Additionally, another dog was reported to have caused the deaths of two dogs within the household.

**Table 4:** Types of wounds inflicted in skin-breaking bites to dogs or other animals.

Injury type	Count	Percent
1–4 small punctures from a single bite.	174	29.4
1–3 small tears or lacerations (up to ½ inch).	85	14.4
Tears or lacerations greater than ½ inch, or fatty tissue, muscle, etc. exposed.	84	14.3
5–8 punctures from the same incident	70	11.8
More than 3 tears or lacerations from multiple bites in the same incident	58	9.8
Killed the other animal	30	5.1
Crushing injuries or broken bones	24	4.1
9–15 punctures from the same incident	20	3.4
Wounded the other animal badly enough for the animal to require euthanasia	18	3.0
Other	13	2.2
16+ punctures from the same incident	13	2.2
I do not know	3	0.5

Response count = 592.

## 4 Discussion

The Behavioral Euthanasia in Pet Dogs Questionnaire was used to investigate factors associated with behavioral euthanasia. Aggression was the primary problem behavior reported by respondents that led to euthanasia. This is consistent with previous studies that identified aggression as the primary behavior concern that led to owners visiting a veterinary behaviorist or choosing to euthanize (17, 19, 26, 28, 46, 47).

Within the larger category of aggression, aggression toward people was both the most reported problem behavior overall and most likely to be selected as the primary reason for euthanasia. This was consistent with the findings of Anderson et al. (17), which showed the majority of aggression cases in a particular behavior clinic were human-directed aggression. This may be due to the safety risks involved with aggression to humans, or might indicate a prioritization of our own species’ safety (75, 76). When only one category of human-directed aggression was reported, aggression toward adults living in the home was the most frequently selected option. At behavior specialty clinics, owner-directed aggression is a commonly-identified type of human-directed aggression exhibited by dogs (23, 26, 53). This might suggest increased difficulty managing aggression toward household members vs. a visitor who is present less often. Aggressing toward household members might also contribute to breaking the human-animal bond resulting in a behavioral euthanasia decision (30, 41, 52, 77).

Aggression toward children was reported less often than aggression toward adults and was rarely selected as the sole target of the dog’s aggression. This is contrary to national dog bite data reporting that children are more frequently taken to the emergency department for dog bites than adults (33). However, it is also possible that post-bite treatment is more regularly sought for children due to the prevalence of bites to the face or higher bite severity to children (33, 78). Children familiar with and living with the dog were selected more frequently than unfamiliar children. This aligns with previous research which indicates that most pediatric bites were caused by a dog familiar to the child (79). While bites or aggression toward children occur frequently according to dog bite statistics, they do not appear to be a common reason for behavioral euthanasia unless combined with other behavior problems. This area warrants future research due to the serious physical and mental health ramifications of bites to children.

When considering the frequency and severity of dog bites, most of the dogs reported to have shown aggression toward people had bitten someone and broken skin. About two-thirds of those dogs had bitten up to three times, but the remaining dogs (36%) had bitten four or more times. While the number of bites alone is a strong indicator for overall behavioral concerns, the severity of the bites might also greatly influence potential euthanasia decisions. While many participants indicated that their dog had inflicted at least one minor bite of one to four small punctures, a substantial portion of responses indicated more severe bites (including those involving many punctures or large tears or lacerations, and those requiring extensive medical treatment). These suggest a heightened public safety risk and impacts on individuals and communities by way of injuries and medical expenses (28, 80). These findings are also consistent with previous research indicating that a history of bites, particularly severe bites, was a significant risk factor for behavioral euthanasia (52, 53).

Aside from human-directed aggression, aggression toward other animals was selected second overall to aggression toward people. Aggression toward other dogs was selected significantly more often than aggression toward non-dog animals. Aggression toward other dogs living in the same household was slightly more frequently reported than toward unfamiliar dogs (31% vs. 28%). However, when participants indicated that their dog only aggressed toward one type of target, other dogs in the home were the sole target more than twice as often as unfamiliar dogs (66% vs. 27%). This might be because dogs living in multi-dog households have more opportunity for intraspecific conflict than those that only encounter other dogs outside the home. This highlights the crucial need for a dog to safely coexist with humans and other dogs in their living environment, as there are serious consequences when they do not.

Aggression toward other dogs was reported more than three times as often as aggression toward other species. Unfamiliar cats, wildlife, and livestock were never the sole target of dog’s animal-directed aggression in this survey, and familiar cats living in the home were rarely marked as the sole target of the dog’s aggression. This might suggest that aggression toward non-dog animals is considered a less serious problem behavior and less likely to result in behavioral euthanasia. Aggression toward non-dog animals is even less likely to result in behavioral euthanasia if the other animals do not share the same household.

For dogs whose owners reported that they displayed aggression toward other animals, a majority of them had bitten another animal and broken skin at least once. Of the dogs who had caused injury to another animal, a significant percentage had done so multiple times (80.9%) and 10.9% had killed or fatally wounded the other animal in one or more incidents. This suggests that owners typically managed and/or sought to resolve this problematic behavior over extended periods of time and after damaging incidents, instead of hastily considering behavioral euthanasia decisions.

Predictability and the presence of warning signals are both considered prognostic factors for aggression risk, and may be useful in evaluating risk of behavioral euthanasia, but have not been objectively assessed (81, 82). Respondents indicated more confidence in predicting the situation or event that could trigger their dog’s aggression than in identifying behaviors that serve as precursors to aggression (e.g., warning signals) in the moment, although participants ranged from “always” to “never” in both predicting aggressive incidents and identifying warning signals. This supports recent work showing that misinterpreting dog behavior can contribute to increased incidents of aggression toward people (83). However, over one-third of respondents indicated being able to predict aggressive incidents most or all of the time, indicating that even with long-term, known aggression issues, perceived predictability of aggression is not enough to prevent behavioral euthanasia for aggression.

Experiencing more than one problem behavior was commonly reported by owners that euthanized a dog for behavior. A large percentage of respondents (80.3%) selected and ranked up to three separate problem behaviors as contributing to their behavioral euthanasia decision for their dog. The data indicate that owners commonly face more than one problem behavior. The most frequently reported comorbidities were aggression toward people and aggression toward other animals. Other studies have also found that many owners reported comorbid behavior problems (23, 24, 48, 84).

Non-aggressive problem behavior (s) were also selected as comorbid factors influencing a behavioral euthanasia decision. Non-aggressive behaviors were most likely to be in combination with aggressive behavior toward humans or other animals, and listed as the second or third behavioral factor contributing to a euthanasia decision. The prominence of aggression over non-aggressive behaviors emphasizes that problem behaviors that increase risk to others, especially risk to humans, are overwhelmingly more influential in behavioral euthanasia decisions than problem behaviors that only pose welfare concerns for the individual dog. This conflicts with recommendations from professionals to consider poor behavioral quality of life as a reason for euthanasia (58, 85).

The results from the Behavioral Euthanasia in Pet Dogs survey indicate that behavioral euthanasia can occur at any age during a dog’s life. Previous studies of behavioral euthanasia have focused on dogs up to 3 years of age (43, 49, 55). However, results from our survey indicated that behavioral euthanasia is not limited to young dogs and can occur in dogs of all ages, from puppies to senior dogs. Additional research is required to understand how factors such as age-related issues or physical or cognitive decline may complicate the ability to manage problem behavior. In the future, researchers exploring behavioral euthanasia should expand their samples to include dogs of all ages.

Finally, most dogs in the sample population lived in their homes for at least 1 year or more, displayed problem behavior (s) for over 1 year, and were involved in multiple aggressive incidents resulting in injury prior to owners electing behavioral euthanasia, indicating that owners made these decisions over time and with extensive consideration for safety.

### 4.1 Limitations and future directions

The Behavioral Euthanasia in Pet Dogs questionnaire was designed to collect data from owners of dogs that were euthanized due to behavior concerns. While the sample used a limited time frame for the euthanasia, recall bias still impacts the retrospective data. Future research should be conducted to assess the reliability and validity of the survey instrument. Additional research could address the recall bias limitation by working with veterinary clinics to provide the survey to owners nearer to the time of behavioral euthanasia rather than retrospectively.

Additionally, the mode of recruitment might have produced a biased sample. The online survey distribution restricted potential respondents to those who had internet access via computer or mobile phone. Researchers chose to recruit a purposive, self-selecting sample of respondents to begin exploring this topic, which may have resulted in a highly motivated sample that was primarily white, female, and highly educated. This bias could be mitigated with future research, for example in-depth interviews of behavior professionals and quantitative study of the prevalence of behavioral euthanasia in veterinary clinics.

However, dog ownership, human-animal relationships, and perceptions of dogs varies greatly by geography, culture, religion, and other societal factors (86–90). Cross-cultural research would uncover similarities and differences in the non-behavioral factors influencing behavioral euthanasia decisions across cultures. Factors like housing, source of the dog, access to animal behavior professionals, implementation of treatment interventions, and non-behavioral factors may also contribute to a behavioral euthanasia decision and should be considered for analysis in future studies.

This paper only addresses behavioral euthanasia in pet dogs; future research should also focus on other species, allowing for comparisons of behavioral euthanasia decisions across those species. Additionally, evaluating the impact of environments (e.g., animals in shelters, foster homes, or even in laboratories), and other life course factors (e.g., owner parental status or life changes such as deaths in the household or moving to a new home) on behavioral euthanasia decisions would help investigate the multifactorial nature of this issue. Future research using in-depth interviews of owners who made the decision to euthanize will help provide clarity and detail about the influence of living situation and non-behavioral factors on behavioral euthanasia decisions.

Research into behavioral euthanasia is understandably challenging. Assessing the incidence, prevalence, change over time, geography, or other factors of behavioral euthanasia in animals is difficult to explore given the lack of centralized reporting of causes of death for the veterinary field similar to reporting in the sheltering industry (e.g., Shelter Animals Count) or vital records (e.g., death certificates for humans) that provides a means to identifying when and why animals were euthanized for behavior (39, 91, 92). With some previous studies estimating that behavior could be the reason for up to one third of all euthanasia in dogs ages three and under, this topic should be further studied in detail (43, 55).

The current study focused on problem behaviors in pet dogs that contribute to the behavioral euthanasia decision by pet dog owners. Research focused on the human experience of the behavioral euthanasia decision process and the impact on the lives and mental health of the remaining family and owner, and the social stigma experienced by humans who made behavioral euthanasia decisions could result in de-stigmatizing this topic and increase the availability of psychosocial support for those grieving after experiencing a behavioral euthanasia (93). Both quantitative surveys and in-depth interviews can help better understand the emotional impacts of behavioral euthanasia on owners, caregivers, and professionals (such as shelter workers, trainers, and veterinarians).

This study is not generalizable to all behavioral euthanasia decisions for pet dogs. Results indicate that multiple factors might contribute to a behavioral euthanasia decision; therefore, individual criteria should not be interpreted to predict behavioral euthanasia decisions for individual pet dogs. Rather, as an exploratory survey, it was intended to uncover insights about a little-researched topic to drive future research design. Future research could look for matched cohorts of dogs with the same problem behavior or combinations thereof that were not euthanized to understand non-behavioral factors that influence behavioral euthanasia decisions.

Additional data from the Behavioral Euthanasia in Pet Dogs Questionnaire could be analyzed to provide insights into the interventions or treatment options pursued by owners prior to behavioral euthanasia, as well as commonalities in factors like housing, source of the dog, and non-behavioral factors that led to the euthanasia decision.

## 5 Conclusion

The Behavioral Euthanasia in Pet Dogs survey is the first exploratory project seeking to understand how problem behaviors in dogs are associated with an owner’s decision to euthanize their dog for behavior concerns. Aggression toward people, followed by aggression toward other animals are the main drivers of behavioral euthanasia in this sample. Safety risks to our own species and to those of any species living in the same household as the dogs who were euthanized were paramount in the decision process. Understanding the behavioral factors that contribute to behavioral euthanasia decisions by owners can help direct additional resources toward successful problem behavior interventions. Improving public education on dog behavior has the potential to strengthen the bond between humans and animals, enhance the quality of life for both, and reduce safety risks in households and communities with multiple species. This, in turn, could lead to a decrease in behavioral euthanasia for pet dogs.

## Data availability statement

The datasets presented in this study can be found in online repositories. The names of the repository/repositories and accession number(s) can be found at: Virginia Tech Data Repository at https://doi.org/10.7294/25222304.

## Ethics statement

The studies involving humans were approved by Virginia Tech Institutional Review Board. The studies were conducted in accordance with the local legislation and institutional requirements. The participants provided their written informed consent to participate in this study.

## Author contributions

MH: Conceptualization, Data curation, Investigation, Methodology, Project administration, Writing – original draft, Writing – review & editing. MW: Conceptualization, Methodology, Supervision, Writing – original draft, Writing – review & editing. AG: Data curation, Formal analysis, Visualization, Writing – review & editing. AR: Conceptualization, Methodology, Supervision, Writing – review & editing. EF: Conceptualization, Methodology, Supervision, Writing – review & editing.
