# Supplementary material for: Factors associated with behavioral euthanasia in pet dogs
Source: Front Vet Sci. 2024 Apr 17;11:1387076. doi: 10.3389/fvets.2024.1387076 (PMC11091869; doi:10.3389/fvets.2024.1387076)
Supplement: Supplementary file 1 [file Data_Sheet_1.pdf]

Supplementary Material

Factors Associated with Behavioral Euthanasia in Pet Dogs

Miranda Hitchcock*, Miranda K. Workman, Adeline P. Guthrie, Audrey Ruple, Erica N. Feuerbacher

*** Correspondence:** Corresponding Author: mirandah@vt.edu

1 Supplementary Data Sheet

Behavioral Euthanasia in Pet Dogs Questionnaire

Notes in blue are internal labels/section headers and will not appear to participants.

INFORMATION PAGE/CONSENT FORM

You are invited to participate in a research study. This form includes information about the study and contact information if you have any questions.

In order to participate in this study, you must:

- Be over the age of 18
- Have euthanized a pet dog primarily due to behavior problems any time **on or after 1/1/2017**. Note: this is for a dog that you owned, not a dog in a shelter or foster environment.
- Have been living (at the time of euthanasia) in the United States

The purpose of this study is to identify factors related to behavioral euthanasia in pet dogs. Currently there is very little information about pet dogs euthanized for behavior reasons, in part due to social stigma and limited tracking mechanisms. Better data and information may lead to improved treatment options, better prevention methods, and more support for people making this decision.

The survey will start with questions about the dog, and about your living situation at the time of the euthanasia. You will then be asked questions about the dog's behavior, interventions, or treatments that may have been attempted, and the euthanasia decision-making process. You will also be asked details about the dog's behaviors, based on your answers in prior sections.

The study should take approximately 15-30 minutes of your time.

We do not anticipate any risks from completing this study, except for potential psychological stress from answering questions of a difficult or emotional nature. If you need support, you may find information through the University of Tennessee Veterinary Social Work Program here: https://vetsocialwork.utk.edu/pet-loss-support-group-2/

You can choose whether to be in this study or not. If you volunteer to be in this study, you may withdraw at any time without consequences of any kind. You may also refuse to answer any questions you don't want to answer and remain in the study. The investigator may withdraw you from this research if circumstances arise which warrant doing so.

We will do our best to protect the confidentiality of the information we gather from you, but we cannot guarantee 100% confidentiality. Your responses are anonymous, so no one can associate your answers back to you. Please do not include your name or other identifying information in your responses that can identify you.

Researcher: Miranda Hitchcock, *mirandah@vt.edu*

IRB# and Title of Study: 22-311 Factors Associated with Behavioral Euthanasia in Pet Dogs

If you have any questions or concerns about the research, please feel free to contact Miranda Hitchcock, *mirandah@vt.edu*. You are not waiving any legal claims, rights or remedies because of your participation in this research study. If you have questions regarding your rights as a research participant, contact the Virginia Tech HRPP Office at 540-231-3732 (irb@vt.edu).

1. Are you over the age of 18?
    a. Yes/No (if no, terminate survey)
2. Have you euthanized a pet dog primarily due to behavior problems any time on or after January 1st, 2017? Note: this is for a dog that you owned, not a dog in a shelter or foster environment.
    a. Yes/No (if no, terminate survey)
3. Were you living in the United States at the time of the euthanasia?
    a. Yes/No (if no, terminate survey)

We understand that this topic may carry strong emotions, and that every behavioral euthanasia case is different. Please answer the questions honestly based on your situation, to the best of your ability and

memory. Your responses are anonymous, and you may save and continue later if you need to take a break.

If you would like to participate in this survey, click START to begin.

DOG INFORMATION

Please answer the following questions about the dog that was euthanized.

4. What was the dog's sex?
    a. Male
    b. Female
5. Was the dog spayed/neutered?
    a. Yes
    b. No
    c. I don't know
6. Was the dog a pure breed or a mixed breed?
    a. Purebred
    b. Mixed Breed
        i. 3a. If Pure bred above: If purebred, what was the breed? (free text)
        ii. 3b. If Mixed breed above: If mixed breed, what was the breed mix? (free text)
7. What was the dog's adult weight in pounds? (known, estimated, or expected) (free text)
8. Where did you get the dog?
    a. Directly from the dog's breeder
    b. Local shelter, humane society, or rescue organization
    c. Out of state or overseas shelter/rescue organization
    d. Friend/Family/Coworker
    e. Pet store
    f. Online
    g. Found
    h. From a litter I bred
    i. Other _______
9. What was the dog's age when you got him/her? (or your best estimate)
    a. Under 8 weeks
    b. Between 8 and 16 weeks
    c. Between 4 and 6 months
    d. Between 6 and 12 months
    e. Between 1 and 3 years
    f. Between 3 and 7 years
    g. 7 years or older
10. How long did you have this dog?
    a. Less than 1 week
    b. Between 1 and 4 weeks
    c. Between 1 and 3 months
    d. Between 3 and 12 months
    e. Between 1 and 3 years
    f. 3 or more years
11. Is there anything else you'd like to tell us about the dog in general? (There will be more specific questions about behavior later) (free text)

LIVING SITUATION

For the questions below, please answer based on your situation just prior to the euthanasia.

12. Which state did you live in?
    a. State dropdown list for United States
13. What type of home did you live in?
    a. Apartment building
    b. Townhouse
    c. Single-family dwelling
    d. RV, trailer, or other mobile dwelling
    e. Farm
    f. Hotel or other temporary dwelling
    g. Other_____
14. Did you rent or own the home listed above?
    a. Rent
    b. Own
    c. Other______
15. What type of area did you live in?
    a. Urban
    b. Suburban
    c. Rural
16. Not including yourself, how many adults (18 years +) lived in the home?
    a. 0, 1, 2, 3, 4, 5, 6, 7, 8, 9, 10+
17. How many children (under 18 years old) lived in the home?
    a. 0, 1, 2, 3, 4, 5, 6, 7, 8, 9, 10+
    b. 18a. If greater than zero children above What were the ages of the children in the home at the time? (select all that apply)
       i. Under 1 year, 1, 2, 3, 4, 5, 6, 7, 8, 9, 10, 11, 12, 13, 14, 15, 16, 17
18. How many other pets (not including the dog) were living *inside* the home?
    a. Dogs (Number scale 0-7+)
    b. Cats (Number scale 0-7+)
    c. Other _______ (Number scale 0-7+)
    d. 19a. If "Other" nonzero above, If you had other pets living inside the home (other than dogs and cats), what type?
19. What other pets or animals (not including the dog) were living at the home *OUTSIDE* that the dog interacted with?
    a. Dogs
    b. Cats
    c. Small livestock (such as chickens)
    d. Large livestock (such as sheep, cows)
    e. Other _______
20. Where did the dog typically sleep at night?
    a. In the bedroom
    b. Elsewhere in the house
    c. In a garage or barn
    d. Outside
    e. Other _______

21. Is there anything else you'd like to tell us about the dog's living situation prior to euthanasia? (free text)

PRIMARY BEHAVIORS (Use these responses for skip logic to the appropriate details sections)

22. What were the most significant behavioral factors in the euthanasia decision? Please select up to three of the following, with the most significant ranked first. If there was only one (or two), leave the rest blank.
    - Aggression toward people
    - Aggression toward dogs or other animals
    - Separation anxiety or separation-related behaviors
    - Compulsive behaviors
    - Fear, anxiety, or stress-related behaviors
    - Other (destructive behavior, house soiling, or anything else)

INTERVENTIONS

The following questions will ask about any treatments, training, or other strategies you may have attempted prior to euthanasia.

26. Did you speak to a veterinarian about the behavior?
    a. Yes
    b. No
    c. 26a. If yes to 26: What veterinarian recommended strategies were attempted? (select all that apply)
        i. Physical examination
        ii. Bloodwork or other diagnostic tests
        iii. Referral to a Veterinary Behaviorist
        iv. Situation behavioral medication (medication given only for specific stressful events or situations)
        v. Daily behavioral medication (medication given at least once per day for anxiety or other problem behavior)
        vi. Supplements, probiotics, or other non-prescription calming aids
        vii. CBD products
        viii. Change in diet
        ix. Other_____
27. Did you attempt any training or behavior modification to address the behavior concerns?
    a. Yes

b. No
c. 27a. If yes to 27, Did you talk with a trainer, behavior consultant, or other dog behavior expert?
    i. Yes
    ii. No
    iii. No, but I am a professional dog trainer or other dog behavior professional
    iv. 27aa. If yes to 27: What types of dog behavior expert(s) did you talk with? (select all that apply)
        1. Trainer
        2. Behavior consultant (a trainer/behaviorist who specifically works with behavior cases)
        3. Veterinary Behaviorist
        4. I am a dog trainer or other dog behavior professional
        5. Other ___________
    v. 27ab. If yes to 27: How many dog behavior experts did you talk with?
        1. Number _____
d. 27b. If yes to 27, What types of training equipment/techniques did you use? (select all that apply) This list of options was randomized
    i. Prong collar or pinch collar
    ii. Choke collar
    iii. Clicker
    iv. Toys
    v. Counter conditioning and desensitization
    vi. Electronic collar, e-collar, or shock collar
    vii. Bark collar or ultrasonic device
    viii. Slip lead
    ix. Squirt bottle
    x. Shake can/penny can
    xi. Citronella spray, "pet corrector", or other similar sprays
    xii. Treats
    xiii. Verbal praise
    xiv. Verbal corrections
    xv. Head halter/gentle leader
    xvi. Alpha/energy
    xvii. Other _______________
e. 27c. If yes to 27, What type of training did you do? (select all that apply)
    i. Private Training
    ii. Group Classes
    iii. Board and Train
    iv. Other: ______________

28. Did you attempt any other strategies to deal with the behavior? (select all that apply)
    a. None
    b. Crates
    c. Basic obedience training
    d. Baby gates, or exercise pens ("ex pens")
    e. White noise, radio, TV, or music
    f. Muzzle
    g. Mental enrichment
    h. Decompression walks
    i. Exercise
    j. Supplements, probiotics, or other non-prescription calming aids

   k. CBD products
   l. Change in diet
   m. Adaptil/ DAP (Dog appeasing pheromone) collar, spray, etc
   n. “Thundershirt” or other compression garment
   o. Dog day care or pet sitters
   p. Other ________

29. Did you attempt to re-home the dog?
   a. Yes
   b. No
   c. 25a. If yes to 25, What type(s) of re-homing did you try? (select all that apply)
      i. Breeder
      ii. Rehome to a friend/family/coworker
      iii. Rehome to another home through Craigslist or similar
      iv. Rescue organization
      v. Shelter
      vi. Sanctuary
      vii. Other: __________
30. What was the total estimated cost you incurred from treatment/management and any other aspects related to the problem behavior(s)? (This includes veterinary or medical care, training, equipment, damage repair, legal expenses, and any other expenses you feel are related to the problem behavior). Approximate dollar amount: (free text)
31. Is there anything else you’d like to tell us about interventions or treatments you attempted? (free text)

DECISION-MAKING AND EUTHANASIA

Please answer the following questions about the decision-making process and the euthanasia process.

32. How long did the behavior problem occur from when you first noticed the behavior to euthanasia (or your best estimate)
   a. Less than 1 week
   b. Between 1 and 4 weeks
   c. Between 1 and 3 months
   d. Between 3 and 12 months
   e. Between 1 and 3 years
   f. More than 3 years
33. Was there a specific incident that led to the euthanasia decision?
   a. Yes, it was a single incident.
   b. Yes, there was a final incident, but after a previous incident(s).
   c. No, there was not a specific incident.
34. Who, if anyone, did you consult with to help make this decision? (select all that apply)
   a. No one
   b. Trainer/Behavior Consultant
   c. Veterinarian
   d. Veterinary Behaviorist
   e. Shelter/Rescue
   f. Friend/Family
   g. Breeder

   h. Other ___________

35. How much did the following factors impact the decision to euthanize? On a 1 to 5 scale where 1 is No Impact and 5 is Major Impact
   - The dog's quality of life
   - The dog's medical issue(s)
   - The dog showing characteristics of old age
   - Worsening behavior or lack of progress
   - Health issues of a person/people in the home
   - Expecting a baby/child
   - Quality of life for the people in the home
   - Individuals in the home with special needs
   - Incompatible with your housing situation, home layout, or physical environment
   - Upcoming travel
   - Upcoming visitors
   - Moving to a new home
   - HOA, landlord, neighbors or other housing restrictions or conflict
   - Safety of people in the home
   - Safety of animals in the home
   - Safety of people/animals outside the home/in the community
   - Legal designation of the dog (for example, Dangerous Dog, Vicious Dog)
   - Fear of legal issues or liability
   - Other legal issues (for example, lawsuits)
   - Financial limitations
   - Inability to rehome
   - Were there other factors that impacted the decision to euthanize? (free text)
36. When did the euthanasia occur?
   a. Month, Year (drop down menus)
37. What was the dog's age at euthanasia in YEARS? (If under a year, please enter "0")
   a. ___
38. Where did the euthanasia occur?
   a. Your regular veterinary clinic (the place you usually get your dog's primary medical care)
   b. Veterinary Behaviorist clinic
   c. A different veterinary clinic or hospital from where you usually get your dog's primary medical care (such as an emergency or specialty clinic)
   d. Your home or property
   e. Location away from home (such as the property of a friend or relative, or a park)
   f. Animal shelter
   g. Other ____________
39. Is there anything else you'd like to tell us about the decision-making and euthanasia process? (free text)

BEHAVIOR DETAILS (Note: Skip logic based on answers in PRIMARY BEHAVIORS. Only see these questions based on what they indicated.)

We would like to better understand your experience and the specific behaviors that led to the decision to euthanize. The sections you see here are based on your responses to the previous question ranking the most significant behavior factors:

- Aggression toward people
- Aggression toward dogs or other animals
- Separation anxiety or separation-related behaviors
- Compulsive behaviors
- Fear, anxiety, or stress
- Other (destructive issues, house soiling, anything else)

Aggression Toward People

Please answer the following about the dog's aggression toward people.

40. Who did the dog demonstrate aggression toward? Select all that apply.
    a. Adults living in the home
    b. Familiar adults not living in the home
    c. Unfamiliar adults
    d. Children living in the home
    e. Familiar children not living in the home
    f. Unfamiliar children
    g. Veterinary staff, groomers, or similar
    h. Other _________
41. How often could you predict situations where the dog would show aggression toward people? (for example, you knew which situations were likely to cause an aggressive response)
    a. Always
    b. Most of the time
    c. About half the time
    d. Once in a while
    e. Never
42. In which of the following circumstances would your dog demonstrate aggression toward people? Select all that apply.
    a. I don't know/didn't see
    b. Person moving quickly or erratically
    c. Being approached while eating
    d. Being approached while playing with/chewing on a favorite toy, bone, or other object
    e. Being approached in/on a preferred space, such as a couch or bed
    f. Having someone approach their preferred person
    g. Being touched, handled, or picked up
    h. Being groomed, bathed, or examined (for things like veterinary care or nail trims)
    i. When an unfamiliar person approached while walking on leash
    j. When mailmen or other delivery workers approached the home
    k. Being startled while sleeping or resting
    l. When a person intervened in a dog fight
    m. A "redirection" where the dog was reacting to something else but bit the person instead, for example when the dog was reacting on leash or running along the fence

n. When scolded or corrected, or given a command
o. During an escalation in play/excitement
p. When the dog was injured or sick
q. None of the above
r. Other _________

43. How often were there clear warning signals leading up to aggressive incidents toward people? (for example, you were able to see signs like growling, ears pinned, or other body language that indicated an aggressive response was coming)
    a. Always
    b. Most of the time
    c. About half the time
    d. Once in a while
    e. Never
44. Did the dog ever demonstrate aggression to people without making contact with its teeth? (for example, lunging and snapping but not actually biting?)
    a. Yes
    b. No
        i. 45a. If yes to 45, how many times (or your best estimate) _____
45. Did the dog ever bite a person and **make contact** with its mouth **without breaking skin**? (for example, leaving bruises but not drawing blood or leaving punctures?
    a. Yes
    b. No
        i. 46a. If yes to 46, how many times (or your best estimate) ____
46. Did the dog ever **bite** a person and **break skin** (for example, drawing blood or leaving punctures)?
    a. Yes
    b. No
        i. 47a. If yes to 47, how many times (or your best estimate) ______
        ii. 47b. If yes to 47, Select the closest description for the bite wound(s). If there were multiple bite incidents, select all that apply.
            1. I don't know
            2. 1-4 small punctures from a single bite.
            3. 5-8 punctures from the same incident
            4. 9-15 punctures from the same incident
            5. 16+ punctures from the same incident
            6. 1-3 small tears or lacerations (up to ½ inch).
            7. More than 3 tears or lacerations from multiple bites in the same incident
            8. Tears or lacerations greater than ½ inch, or fatty tissue, muscle, etc exposed.
            9. Crushing injuries or broken bones
            10. Other __________
        iii. 47c. If yes to 47 What medical treatment (if any) was needed after the bite(s)? (select all that apply)
            1. I don't know
            2. None
            3. Treated at home (including cleaning, bandaging, over-the-counter medication)
            4. Treated at a doctor's office
            5. Treated at urgent care
            6. Treated at emergency center/emergency room
            7. Ambulance
            8. Admitted at the hospital (usually at least overnight)
            9. 1-10 stitches
            10. 11-20 stitches

11. 21+ stitches
12. Other surgery required
13. Other______

iv. 47d. If yes to 47 On which part of the person's body did the bites occur? (select all that apply)
1. I don't know
2. Hands or arms
3. Feet or legs
4. Face
5. Trunk (groin, buttocks, abdomen, chest, shoulders, or back)
6. Neck and back of the head
7. Other: _____

v. 47e. If yes to 47 Did any factors (potentially) lessen the severity of any of the the bite incident(s)? (select all that apply)
1. No
2. Sweatshirt, jacket, heavy clothing
3. Boots or shoes
4. Muzzle
5. Intervention from a person
6. Other _______

vi. 47f. If yes to 47 Did the dog have to overcome any barriers to bite in any of these incidents? (select all that apply)
1. No
2. Jumped/broke a fence
3. Jumped/broke a baby gate
4. Crossed the room/area from at least 10 feet away
5. Broke a collar or leash
6. Dragged the handler on leash
7. Other ________

47. Is there anything else you'd like to tell us about the aggression toward people? (free text)

-

-
- **Aggression Toward Dogs or Other Animals**

Please answer the following about the dog's aggression toward other animals.

-

48. What type of animal did the dog demonstrate aggression toward? (select all that apply)
   a. Other dogs in the home
   b. Familiar/known dogs not living in the home
   c. Unfamiliar dogs
   d. Cats in the home
   e. Unfamiliar cats
   f. Other: _____________
49. How often were you able to predict situations where the dog would show aggression toward other animals? (for example, you knew which situations were likely to cause an aggressive response)
   a. Always
   b. Most of the time
   c. About half the time
   d. Once in a while
   e. Never

50. What circumstances or triggers were involved in aggression incidents toward other animals? Select all that apply.
    a. I don't know
    b. Being approached while eating
    c. Being approached while playing with/chewing on a favorite toy, bone, or other object
    d. Being approached in/on a preferred area, such as a couch or bed
    e. When a preferred person was approached by the other animal
    f. Being approached directly by the other animal while the dog was on leash
    g. When an animal entered the dog's yard or home
    h. When woken up from sleeping or startled
    i. A "redirection" where the dog was reacting to something else but bit the other animal instead, for example when the dog was reacting on leash or running along the fence
    j. Predatory behavior (attempting to hunt or prey on the other animal)
    k. Joined in when other dog(s) were already fighting
    l. The other animal attacked, cornered, or otherwise threatened the dog
    m. Escalation in play/excitement
    n. Changes in health or appearance of the other animal
    o. The dog itself was injured, sick, etc
    p. None of the above
    q. Other _____________
51. How often were there clear warning signals leading up to aggressive incidents? (for example, you were able to see signs like growling, ears pinned, or other body language that indicated an aggressive response was coming)
    a. Always
    b. Most of the time
    c. About half the time
    d. Once in a while
    e. Never
52. Did the dog ever demonstrate aggression **without making contact** with its teeth? (for example, lunging and snapping but not actually biting?)
    a. Yes
    b. No
        i. 53a. If yes to 53 How many times (it's ok to estimate) _____
53. Did the dog ever bite and **make contact** with its mouth **without breaking skin**? (for example, leaving bruises but not drawing blood or leaving punctures?
    a. No
    b. Yes
        i. 54a. If yes to 54 How many times (it's ok to estimate)
54. Did the dog ever **bite** another dog or animal and **break skin**?
    a. No
    b. Yes
        i. 55a. If yes to 55 If yes, how many times (It's ok to estimate)
        ii. 55b. If yes to 55 Please select the closest description for the bite wound(s). If there were multiple bite incidents, select all that apply.
            1. I don't know
            2. 1-4 small punctures from a single bite.
            3. 5-8 punctures from the same incident
            4. 9-15 punctures from the same incident
            5. 16+ punctures from the same incident
            6. 1-3 small tears or lacerations (up to ½ inch).
            7. More than 3 tears or lacerations from multiple bites in the same incident

8. Tears or lacerations greater than ½ inch, or fatty tissue, muscle, etc exposed.
9. Crushing injuries or broken bones
10. Killed the other animal
11. Wounded the other animal badly enough for the animal to require euthanasia
12. Other __________

iii. 55c. If yes to 55 What veterinary treatment (if any) was needed after the bite(s)? (select all that apply)
1. I don't know
2. None
3. Treated at home (including cleaning, bandaging, over-the-counter medication)
4. Wound care at a veterinary clinic
5. 1-6 stitches
6. 7-20 stitches
7. 21+ stitches
8. Surgery required
9. Other animal died or was euthanized due to its injuries

iv. 55d. If yes to 55 On which part of the body was the animal bitten? Select all that apply.
1. I don't know
2. Front leg(s) or shoulder
3. Hind leg(s) or hips
4. Snout/muzzle/face/head/ears
5. Back or side of the body
6. Abdomen or belly
7. Tail
8. Neck
9. Chest
10. Other

v. 55e. If yes to 55 Did any factors (potentially) lessen the severity of any of the the bite incident(s)? (select all that apply)
1. No
2. Thick fur
3. Other animal wearing harness, sweater, blanket, or other
4. Intervention from a person
5. Muzzle
6. Other ______

vi. 55f. If yes to 55 Did the dog have to overcome any barriers to bite in any of these incidents? (select all that apply)
1. No
2. Jumped/broke a fence
3. Jumped/broke a baby gate
4. Crossed the room/area from at least 10 feet away
5. Broke a collar or leash
6. Dragged the handler on leash
7. Other _______

55. Is there anything else you'd like to tell us about the aggression toward animals? (free text)

Separation Anxiety or Separation-Related Behaviors

•

Please answer the following questions about the dog's separation anxiety or separation-related behaviors.

•

56. How often did the dog show each of the following problem behaviors or signs of anxiety when left, or about to be left, alone? (Alone means being left in an area without people, though another dog might be present). Scale from left to right of Never, Once in a while, About half the time, Most of the time, Always
    a. Restlessness/agitation/pacing
    b. Barking or whining
    c. Chewing/scratching at doors, floor, windows, curtains, etc
    d. Urination/defecation
    e. Self-harm (for example, from licking or scratching)
    f. Trembling or shaking
    g. Hiding
    h. Escaping confinement (such as a crate)
    i. Escaping the area (such as a door or window)
57. Were there other signs of anxiety or separation-related behavior that the dog showed when left alone? (free text)
58. Where was the dog left alone when they showed the anxiety or separation-related behavior? (select all that apply)
    a. In the house (full access)
    b. In a specific room or area gated off
    c. In a crate
    d. Outdoors
    e. In a garage or barn (or similar)
    f. Other____________
59. Did the dog demonstrate signs of distress when left with someone other than their "preferred" person?
    a. Yes
    b. No
    c. Sometimes
    d. I don't know
60. During these behaviors, did the dog ever injure itself enough to need veterinary care?
    a. Yes/No
    b. 61b If yes, how many times? (it's ok to estimate)
61. Is there anything else you'd like to tell us about the separation anxiety or separation related behavior? (free text)

Compulsive Behaviors

Please answer the following questions about the dog's compulsive behaviors.

•

62. Which repetitive or compulsive behaviors did the dog exhibit (select all that apply)
    a. Star gazing (staring at the sky or ceiling)
    b. Light or shadow chasing
    c. Tail chasing or spinning
    d. Licking
    e. Sucking

f. Path-running or pacing
g. Fly biting/ fly snapping (staring at or snapping at the air)
h. Other ___________

63. How frequently did the dog demonstrate compulsive behaviors?
    a. Monthly
    b. Weekly
    c. Once per day
    d. Multiple times per day (2-9 times)
    e. 10+ times per day
64. On average, how long did the behavior last?
    a. Less than one minute
    b. 1-5 minutes
    c. 6-30 minutes
    d. More than 30 minutes
65. During these behaviors, did the dog ever injure itself enough to need veterinary care?
    a. Yes/No
    b. 66a. If yes to 66, How many times? (it's ok to estimate) (free text)
66. How frequently did the compulsive behaviors disrupt the dog's ability to engage in healthy behaviors such as eating, playing, and sleeping?
    a. Always
    b. Most of the time
    c. About half the time
    d. Once in a while
    e. Never
    f. N/A
67. Is there anything else you'd like to tell us about the compulsive behavior? (free text)

•

Fear, Anxiety, or Stress-Related Behaviors

Please answer the following questions about the dog's fear, anxiety, or stress. When answering this set of questions, please do not include separation anxiety.

•

68. How severe was the dog's fear, anxiety, or stress toward the following. (please do not include separation anxiety in this section). (on a scale of 1-5, with 1 being no fear, and 5 being extreme fear.) An N/A response is included
    a. Sudden noises
    b. Unfamiliar people
    c. Unfamiliar dogs
    d. Familiar people
    e. Familiar dogs
    f. Being outside
    g. New locations/situations
    h. New objects/items
    i. Passing vehicles
    j. Riding in the car or other vehicle
    k. Veterinarian or veterinary clinic
    l. Surfaces/textures (such as floor types)
69. Were there other circumstances or situations that caused fear, anxiety, or stress for the dog? (free text)
70. How often were you able to predict situations where the dog would show fear, anxiety, or stress?
    a. Always

   b. Most of the time
   c. About half the time
   d. Once in a while
   e. Never
71. What behaviors did the dog display that indicated fear, anxiety, or stress? (select all that apply)
   a. Running away or into another room
   b. Cowering
   c. Hiding behind/under objects
   d. Panting
   e. Drooling
   f. Whining/crying
   g. Barking
   h. Trembling
   i. Freezing/immobility
   j. Pancaking (lying on the floor without moving)
   k. Other
72. How often did you observe signs of fear, anxiety, or stress? (on average, it's ok to estimate)
   a. Monthly
   b. Weekly
   c. Once per day
   d. Multiple times per day (2-9 times)
   e. 10+ times per day
73. On average, per episode, how long did the fear, anxiety, or stress last?
   a. Less than one minute
   b. Between 1 and 5 minutes
   c. Between 6 and 30 minutes
   d. More than 30 minutes
   e. I don't know
74. During these behaviors, did the dog ever injure itself enough to need veterinary care?
   a. Yes/No
   b. 75a. If yes to 75, How many times? (it's ok to estimate) (free text)
75. Is there anything else you'd like to tell us about the dog's fear, anxiety, or stress? (free text)
   •

Other (Destructive Behavior, House Soiling, Anything Else)

•

76. Please describe the other behaviors that contributed to the decision to euthanize. (free text)

ALL participants go here, regardless of which primary behaviors they chose

77. Is there anything else you'd like to tell us about the behaviors or other factors that influenced the euthanasia decision? (free text)

DEMOGRAPHICS

In order for us to better understand our survey population, please complete the following information.

78. What is your gender identity (select all that apply)
    a. Woman
    b. Man
    c. Transgender
    d. Non-binary/non-conforming
    e. Other
    f. Prefer not to respond
79. What is your race (select all that apply)
    a. White
    b. Black or African American
    c. American Indian or Alaska Native
    d. Asian
    e. Native Hawaiian or Other Pacific Islander
    f. Other
    g. Prefer not to respond
80. Are you of Hispanic, Latino, or Spanish origin?
    a. No
    b. Yes
    c. Prefer not to respond
81. What was your age at the time of the dog's euthanasia?
    a. 18- 24 years old
    b. 25- 34 years old
    c. 35- 44 years old
    d. 45- 54 years old
    e. 55- 64 years old
    f. 65+ years old
    g. Prefer not to respond
82. What was the highest level of education you had completed at the time of the dog's euthanasia?
    a. Some high school
    b. High school diploma or GED
    c. Some college
    d. Associate's degree
    e. Bachelor's degree
    f. Master's degree
    g. Professional degree beyond bachelor's degree (for example, MD, JD, DVM)
    h. Doctorate (for example, PhD)
    i. Prefer not to respond
83. What was your annual household income range at the time of the dog's euthanasia?
    a. Less than $20,000
    b. $20,000 to $34,999
    c. $35,000 to $49,999
    d. $50,000 to $74,999
    e. $75,000 to $99,999
    f. $100,000 to $199,999
    g. $200,000 or more
    h. Prefer not to respond
84. How did you find out about this research?

a. Losing Lulu Facebook Page
b. Other dog-related social media group
c. Veterinarian
d. Trainer/behavior professional
e. Friend/family/coworker
f. Other ____________

Thank you for participating in this research project. We understand that participation may bring up difficult emotions. If you need support, you may find information through the University of Tennessee Veterinary Social Work Program here: https://vetsocialwork.utk.edu/pet-loss-support-group-2/

If you are interested in participating in future research, or in receiving the results of this study, please email at mirandah@vt.edu with the subject line "STUDY FOLLOW UP"
