# Supplementary material for: Factors associated with behavioral euthanasia in pet dogs
Source: Front Vet Sci. 2024 Apr 17;11:1387076. doi: 10.3389/fvets.2024.1387076 (PMC11091869; doi:10.3389/fvets.2024.1387076)

Supplementary Material

Factors Associated with Behavioral Euthanasia in Pet Dogs

Miranda Hitchcock*, Miranda K. Workman, Adeline Guthrie, Audrey Ruple, Erica Feuerbacher

*** Correspondence:** Corresponding Author: mirandah@vt.edu

1 Supplementary Figure 1

Title: Participant Response Count (n) at Different Points in the Behavioral Euthanasia in Pet Dogs Questionnaire

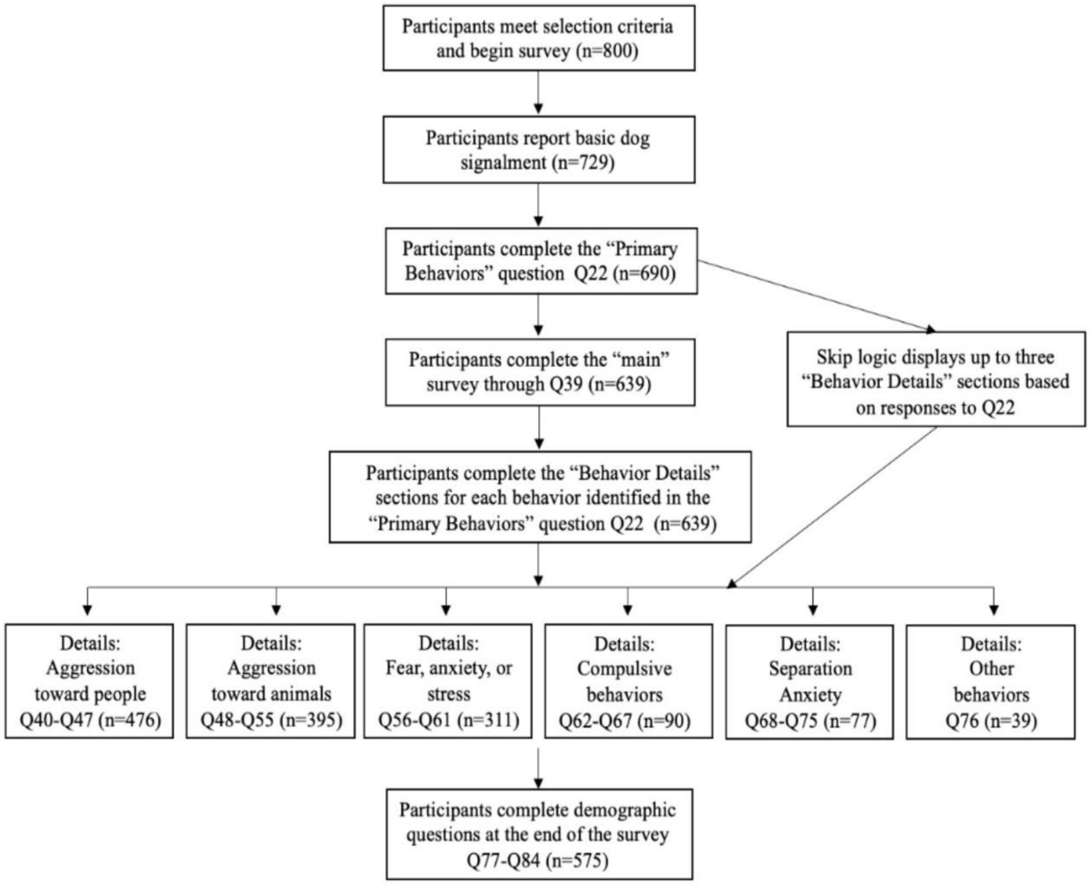

Supplement: Supplementary file 2 [file Image_1.pdf]
